# Redetermination of 1,3,6,8-tetra­aza­tri­cyclo­[4.4.1.1^3,8^]dodeca­ne

**DOI:** 10.1107/S1600536814002608

**Published:** 2014-02-08

**Authors:** Augusto Rivera, Jaime Ríos-Motta, Michael Bolte

**Affiliations:** a Universidad Nacional de Colombia, Sede Bogotá, Facultad de Ciencias, Departamento de Química, Cra 30 No.45-03, Bogotá, Código Postal 111321, Colombia; b Institut für Anorganische Chemie, J. W. Goethe-Universität Frankfurt, Max-von-Laue-Strasse 7, 60438 Frankfurt/Main, Germany

## Abstract

The structure of the title compound, C_8_H_16_N_4_, which consists of four fused seven-membered rings, has been redetermined at 173 K. This redetermination corrects the orientation of two H atoms, which were located at unrealistic positions in the original room-temperature study [Murray-Rust (1974 ▶). *J. Chem. Soc. Perkin Trans. 2*, pp. 1136–1141]. The complete mol­ecule is generated by -42*m* symmetry, with one quarter of a mol­ecule [one N atom (site symmetry *m*), two C atoms (one with site symmetry *m* and the other with site symmetry 2) and two H atoms] in the asymmetric unit. No directional inter­actions beyond van der Waals contacts are apparent in the crystal structure.

## Related literature   

For the original synthesis procedure, see: Bischoff (1898 ▶). For the previous determination of the structure, see: Murray-Rust (1974 ▶). For crystal structures containing the title compound as a fragment, see: Rivera *et al.* (2007 ▶); Glister *et al.* (2005 ▶). For a description of the Cambridge Crystallographic Database, see: Allen *et al.* (2002 ▶).
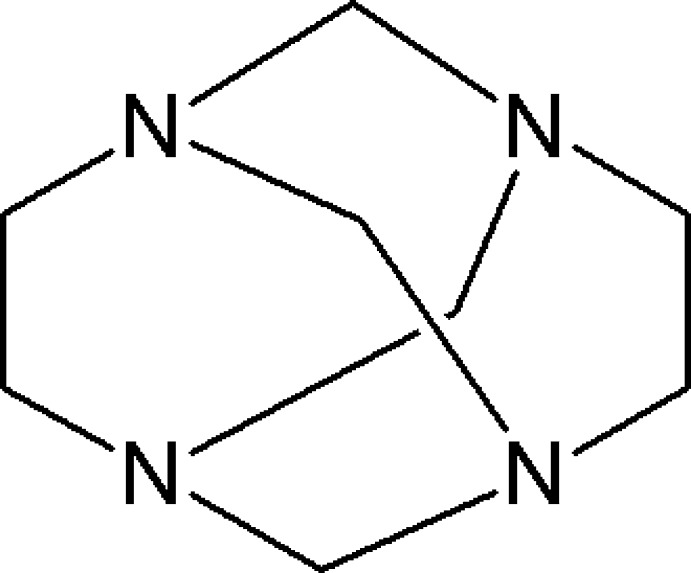


## Experimental   

### 

#### Crystal data  

C_8_H_16_N_4_
*M* *_r_* = 168.25
Tetragonal, 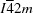

*a* = 7.4065 (7) Å
*c* = 7.6235 (8) Å
*V* = 418.20 (9) Å^3^
*Z* = 2
Mo *K*α radiation
μ = 0.09 mm^−1^
*T* = 173 K
0.32 × 0.28 × 0.27 mm

#### Data collection  

Stoe IPDS II two-circle diffractometer
Absorption correction: multi-scan (*X-AREA*; Stoe & Cie, 2001 ▶) *T*_min_ = 0.973, *T*_max_ = 0.977
4336 measured reflections
264 independent reflections
264 reflections with *I* > 2σ(*I*)
*R*_int_ = 0.049

#### Refinement  

*R*[*F* ^2^ > 2σ(*F* ^2^)] = 0.050
*wR*(*F* ^2^) = 0.141
*S* = 1.12
264 reflections
18 parameters
H-atom parameters constrained
Δρ_max_ = 0.27 e Å^−3^
Δρ_min_ = −0.17 e Å^−3^

### 

Data collection: *X-AREA* (Stoe & Cie, 2001 ▶); cell refinement: *X-AREA*; data reduction: *X-AREA*; program(s) used to solve structure: *SHELXS97* (Sheldrick, 2008 ▶); program(s) used to refine structure: *SHELXL97* (Sheldrick, 2008 ▶); molecular graphics: *XP* in *SHELXTL* (Sheldrick, 2008 ▶); software used to prepare material for publication: *SHELXL97*.

## Supplementary Material

Crystal structure: contains datablock(s) I, New_Global_Publ_Block. DOI: 10.1107/S1600536814002608/hb7192sup1.cif

Structure factors: contains datablock(s) I. DOI: 10.1107/S1600536814002608/hb7192Isup2.hkl

Supporting information file. DOI: 10.1107/S1600536814002608/hb7192Isup3.cml

CCDC reference:

Additional supporting information:  crystallographic information; 3D view; checkCIF report

## Figures and Tables

**Fig. 1.**
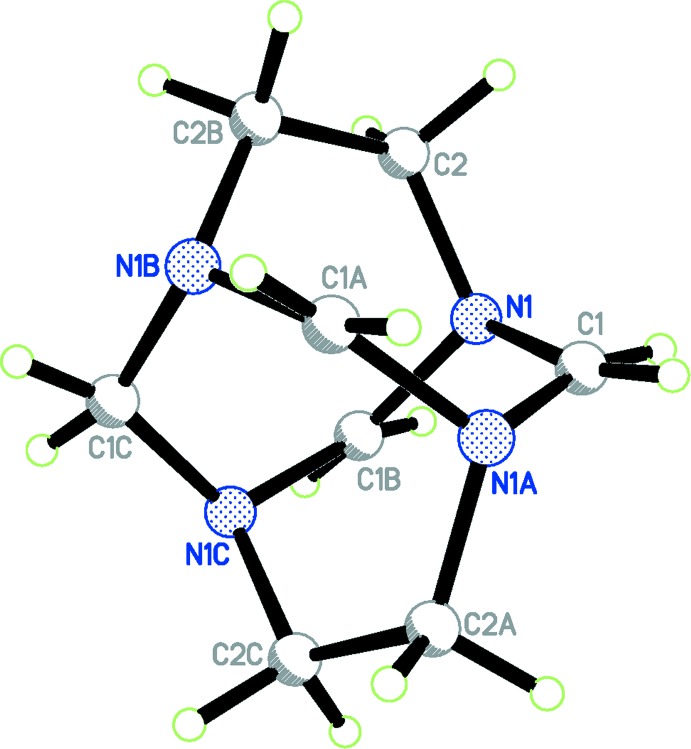
A view of the molecular structure of the title compound as determined by Murray-Rust (1974).

**Fig. 2.**
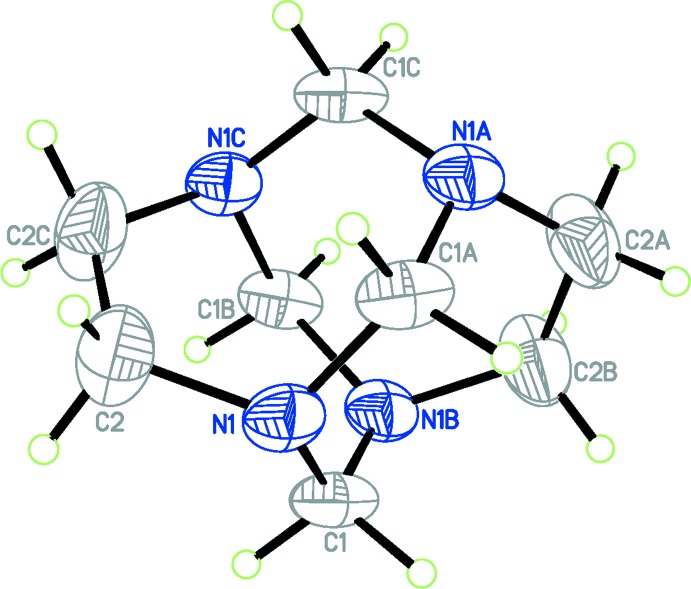
A view of the crystal structure of the title compound with the numbering scheme. Displacement ellipsoids are drawn at the 50% probability level. Symmetry operators for generating equivalent atoms: (A) -y, x, -z, (B) y, -x, -z, (C) -x, -y, z.

**Fig. 3.**
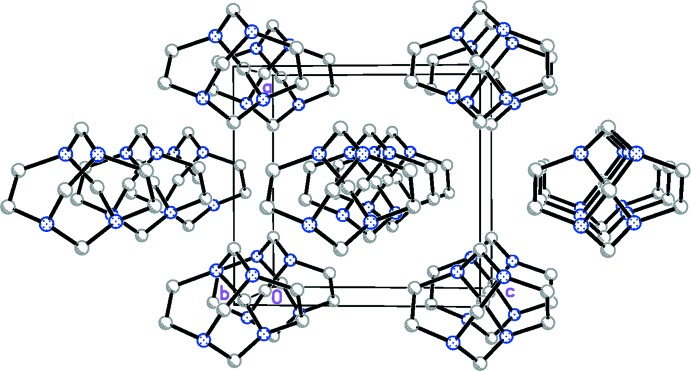
Packing diagram of the title compound with view onto the ac plane.

## supplementary crystallographic information

### 1. Comment

The crystal structure of the title compound, a fully saturated cage-like molecule, was first reported (Murray-Rust, 1974) at room temperature. This heterocyclic system is of considerable conformational interest. While by now the structure of the molecule would seem well established, the position of some hydrogen atoms is rather strange (Fig. 1). Both H atoms bonded to C1 almost lie in a common plane with the two N atoms attached to C1 (r.m.s. deviation for all five atoms 0.050 Å). The dihedral angle between the NC_2_ plane and the CH_2_ plane is 10.7°. However, the CH_2_ plane should be more or less perpendicular to the CN_2_ plane.

Our interest in the title compound prompted the present re-investigation carrying out the data collection at 173 (2) K which provides more regular positions for the H atoms (Fig. 2).

The re-determination of the crystal structure is consistent with the observation that TATD belongs to the *D*_2_*_d_* point group as pointed out Murray-Rust. The only significant difference is the localization of the H atoms. The largest difference between the two determinations pertains to the C—C distance of the ethylene bridge. In the original study this distance was determined as 1.534 (8) Å, whereas it is 1.477 (8) Å in this study. Taking the displacement ellipsoid which is elongated perpendicular to the N—C—C—N moiety into account, the shortened bond length might be due to a slight disorder of C2.

Concerning the H atoms, the orientation of the methylene group connecting two N atoms in particular is corrected: in the structure of Murray-Rust the orientation of the H atoms is such that both C—H bonds are in the same plane with the N—C bonds, with a dihedral angle of 10.7° between the NC_2_ plane and the CH_2_ plane. In the redetermination, the dihedral angle between these planes is exactly 90°.

The title molecule is composed of four seven-membered rings which have exactly the same conformation due to the molecular symmetry. The conformation can be described as a chair. A search in the Cambridge Crystallographic Database (CSD, Version 5.35 of 2013, plus two updates; Allen 2002) yielded three structures containing the title compound as a fragment, namely the structure determination of Murray-Rust (1974), the title compound as a co-crystal with hydroquinone (Rivera *et al.*, 2007), the title compound substituted with a cyclohexane ring at the C—C bonds (Glister *et al.*, 2005). The conformation of the seven-membered rings is a chair in all cases which is not surprising since the the molecule is rigid.

Concerning the crystal packing, molecules of the title compound are located at the origin and at the centre of the unit cell. Thus, the crystal packing can be regarded as two sets of layers. The molecules in neighbouring layers are displaced by the symmetry operation 1/2 + *x*, 1/2 + *y*, 1/2 + *z* (Fig. 3).

### 2. Experimental

1,3,6,8-tetraazatricyclo[4.4.1.1^3,8^]dodecane (TATD) was synthesized from formaldehyde and diaminoethane as described in the literature (Bischoff, 1898) and recrystallized from 1,4-dioxane solution as colourless blocks.

### 3. Refinement

All H atoms were located in a difference map. Nevertheless, they were geometrically placed and refined using a riding model, with C—H = 0.99 Å and with *U*_iso_(H) = 1.2*U*_eq_(C). The absolute structure was indeterminate in the present refinement.

### Crystal data

**Table:**

C_8_H_16_N_4_	*D*_x_ = 1.336 Mg m^−^^3^
*M**_r_* = 168.25	Mo *K*α radiation, λ = 0.71073 Å
Tetragonal, *I*42*m*	Cell parameters from 11628 reflections
*a* = 7.4065 (7) Å	θ = 3.8–27.9°
*c* = 7.6235 (8) Å	µ = 0.09 mm^−^^1^
*V* = 418.20 (9) Å^3^	*T* = 173 K
*Z* = 2	Block, colourless
*F*(000) = 184	0.32 × 0.28 × 0.27 mm

### Data collection

**Table:**

Stoe IPDS II two-circle diffractometer	264 reflections with *I* > 2σ(*I*)
Radiation source: Genix 3D IµS microfocus X-ray source	*R*_int_ = 0.049
ω scans	θ_max_ = 27.5°, θ_min_ = 5.4°
Absorption correction: multi-scan (*X-AREA*; Stoe & Cie, 2001)	*h* = −9→8
*T*_min_ = 0.973, *T*_max_ = 0.977	*k* = −9→9
4336 measured reflections	*l* = −9→9
264 independent reflections	

### Refinement

**Table:**

Refinement on *F*^2^	0 restraints
Least-squares matrix: full	Hydrogen site location: inferred from neighbouring sites
*R*[*F*^2^ > 2σ(*F*^2^)] = 0.050	H-atom parameters constrained
*wR*(*F*^2^) = 0.141	*w* = 1/[σ^2^(*F*_o_^2^) + (0.0809*P*)^2^ + 0.2587*P*] where *P* = (*F*_o_^2^ + 2*F*_c_^2^)/3
*S* = 1.12	(Δ/σ)_max_ < 0.001
264 reflections	Δρ_max_ = 0.27 e Å^−^^3^
18 parameters	Δρ_min_ = −0.17 e Å^−^^3^

### Special details

**Table:**

Geometry. All e.s.d.'s (except the e.s.d. in the dihedral angle between two l.s. planes) are estimated using the full covariance matrix. The cell e.s.d.'s are taken into account individually in the estimation of e.s.d.'s in distances, angles and torsion angles; correlations between e.s.d.'s in cell parameters are only used when they are defined by crystal symmetry. An approximate (isotropic) treatment of cell e.s.d.'s is used for estimating e.s.d.'s involving l.s. planes.
Refinement. ;

### Fractional atomic coordinates and isotropic or equivalent isotropic displacement parameters (Å^2^)

**Table:**

	*x*	*y*	*z*	*U*_iso_*/*U*_eq_	
N1	0.1352 (2)	0.1352 (2)	0.0995 (4)	0.0373 (9)	
C1	0.2345 (4)	0.0000	0.0000	0.0406 (10)	
H1	0.3140	0.0649	−0.0832	0.049*	
C2	0.0705 (4)	0.0705 (4)	0.2690 (5)	0.0604 (14)	
H2	0.0235	0.1756	0.3350	0.073*	

### Atomic displacement parameters (Å^2^)

**Table:**

	*U*^11^	*U*^22^	*U*^33^	*U*^12^	*U*^13^	*U*^23^
N1	0.0317 (10)	0.0317 (10)	0.0483 (16)	−0.0099 (11)	0.0011 (8)	0.0011 (8)
C1	0.0228 (14)	0.0310 (16)	0.068 (2)	0.000	0.000	0.0063 (15)
C2	0.071 (2)	0.071 (2)	0.0392 (17)	−0.038 (2)	−0.0017 (11)	−0.0017 (11)

### Geometric parameters (Å, º)

**Table:**

N1—C1^i^	1.456 (2)	C1—H1	0.9900
N1—C1	1.456 (2)	C2—C2^iii^	1.477 (8)
N1—C2	1.459 (5)	C2—H2	0.9900
C1—N1^ii^	1.456 (2)		
			
C1^i^—N1—C1	115.0 (3)	N1—C1—H1	107.5
C1^i^—N1—C2	113.66 (12)	N1—C2—C2^iii^	117.68 (18)
C1—N1—C2	113.66 (12)	N1—C2—H2	107.9
N1^ii^—C1—N1	119.4 (3)	C2^iii^—C2—H2	107.9
N1^ii^—C1—H1	107.5		
			
C1^i^—N1—C1—N1^ii^	−52.65 (18)	C1^i^—N1—C2—C2^iii^	67.1 (2)
C2—N1—C1—N1^ii^	80.8 (2)	C1—N1—C2—C2^iii^	−67.1 (2)

Symmetry codes: (i) −*y*, *x*, −*z*; (ii) *y*, −*x*, −*z*; (iii) −*x*, −*y*, *z*.
